# Mechanochemistry II

**DOI:** 10.3762/bjoc.15.154

**Published:** 2019-07-09

**Authors:** José G Hernández

**Affiliations:** 1 Institute of Organic Chemistry, RWTH Aachen University, Landoltweg 1, D-52074 Aachen, Germany

**Keywords:** green chemistry, mechanochemistry, methods, organic chemistry

Since the publication of the first thematic issue on mechanochemistry in the *Beilstein Journal of Organic Chemistry* in 2017 [1], the global interest in the field of mechanochemistry has continued exponentially growing. Thus, leading to the implementation of mechanochemical techniques across different areas of science. Such tremendous growth has established mechanochemistry as a sustainable strategy for the future of chemical synthesis. In fact, the potential of mechanochemistry in various domains of research, industry and in commercial entities has been recently recognized by the IUPAC after the inclusion of mechanochemistry among the ten chemical innovations that will change our world [2]. Therefore, once again, the *Beilstein Journal of Organic Chemistry* is contributing to the dissemination of mechanochemistry in the field of organic chemistry through a new thematic issue Mechanochemistry II.

In this new collection of works, the readership of the *Beilstein Journal of Organic Chemistry* will find contributions from renowned global experts in the field of mechanochemistry spanning areas from organic mechanochemistry, supramolecular mechanochemistry to polymer mechanochemistry. Moreover, findings reported in this current thematic issue also contribute to the expansion of synthetic chemistry methodology by mechanochemistry. Importantly, such rapid advancement in applied mechanochemistry is supported by investigations focused on better understanding the fundamental aspects governing mechanochemical transformations.

Therefore, I hope the joint efforts made by the authors of these contributions (review articles, letters, and full research papers), and the *Beilstein Journal of Organic Chemistry* could augment the consolidation of mechanochemistry as an alternative to continue with the development of chemical processes in a more efficient manner.

José G. Hernández

Aachen, June 2019
